# Oxygen Vacancy: How Will Poling History Affect Its Role in Photoelectrocatalysis

**DOI:** 10.1002/cssc.202400946

**Published:** 2024-08-08

**Authors:** Xianlong Li, Zhiliang Wang, Yifan Bao, Haijiao Lu, Jiakang You, Lianzhou Wang

**Affiliations:** ^1^ Nanomaterials Centre School of Chemical Engineering Australian Institute for Bioengineering and Nanotechnology The University of Queensland St Lucia 4072 Queensland Australia

**Keywords:** oxygen vacancy, defect dipole, dipole moment, charge separation and transfer

## Abstract

Oxygen vacancy (V_O_) has been recognized to possess an effect to promote the charge separation and transfer (CST) in various n‐type semiconductor based photoelectrodes. But how external stimulus will change this V_O_ effect has not been investigated. In this work, external polarization is applied to investigate the effect of V_O_ on the CST process of a typical ferroelectric BiFeO_3_ photoelectrode. It is found that negative poling treatment can significantly boost V_O_ effect, while positive poling treatment will deteriorate the CST capability in BiFeO_3_ photoelectrodes. This poling history determined V_O_ effect is rooted in the V_O_ induced defect dipoles, wherein their alignment produces a depolarization electric field to modulate the CST driving force. This finding highlights the significance of poling history in functionalizing the V_O_ in a photoelectrode.

## Graphical abstract

The effect of oxygen vacancy in influencing charge separation and transfer capability depends on the poling history the photoelectrode has experienced, including the poling direction, poling voltage, relaxation duration and so on.

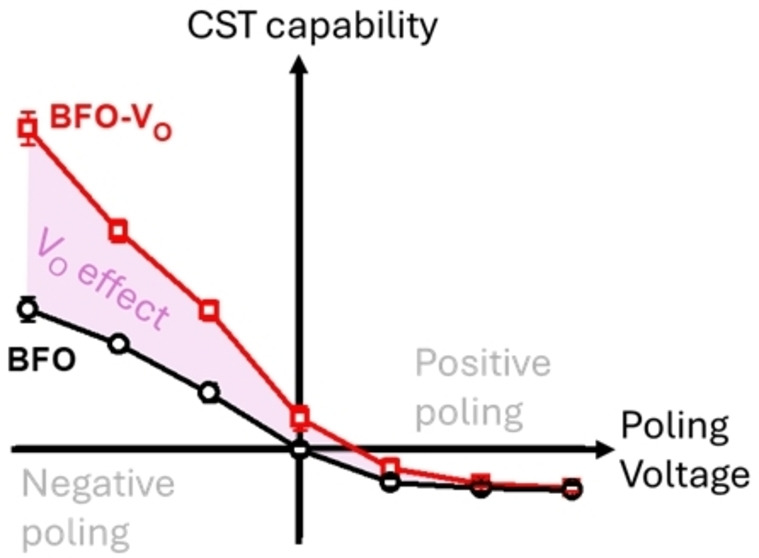

## Introduction

Oxygen vacancy (V_O_) widely exists in different metal oxides due to the intrinsic oxygen deficiency during synthesis.[^1^, ^2^] Due to the charge compensation of V_O_,^[3]^ it endows the n‐type conductivity for many metal oxide semiconductors, which lays the foundation for many significant applications such as blue light emission diode,[^4^, ^5^] field effect transistor[^6^, ^7^] and solar energy conversion.[^2^, ^5^] Especially in the community of solar energy conversion, it is commonly accepted that moderate concentration of V_O_ is beneficial to charge separation and transfer (CST) process of n‐type semiconductors applications, such as photoanodes for photoelectrocatalysis.[^12^, ^13^, ^14^, ^15^] But this knowledge is acquired under classical environment with light irradiation only, how the V_O_ effect will change under external stimulus is still yet to be understood.

As a kind of typical lattice defect, V_O_ can be positively charged and theoretically create defect dipoles in metal oxides,^[16]^ which can change the polarization of a crystal.[^17^, ^18^] In photocatalysts design, polarization has been applied to generate additional electric field for efficient photogenerated charge separation and transfer (CST).[^19^, ^20^, ^21^, ^22^] In our previous research based on ferroelectric BiFeO_3_ photoanodes, we also have revealed that proper alignment of the polarization direction can largely tune the photoresponse in photoelectrodes.^[10]^ Considering that the V_O_ induced defect dipoles may interfere with the intrinsic dipoles in ferroelectric materials,[^24^, ^25^] it raises an interesting question: what is the influence of V_O_ on the performance of ferroelectric photoanodes? Clarifying this question will provide a more comprehensive understanding about the nature of V_O_, providing guide to the application of V_O_ in various fields.

Herein we use BiFeO_3_, a ferroelectric with narrow bandgap and high Curie temperature,^[12]^ as a prototype to gain insight into the mystery of V_O_’s function. The V_O_ concentration is first tailored via conventional heating treatment. Further photoelectrochemical (PEC) investigation shows the influence of V_O_ is dramatically dependent on the poling history. Higher V_O_ concentration results in more profound poling voltage dependent V_O_ effect. In addition, relaxation of aligned dipoles leads to photocurrent decay, which in turn can be fully recovered via another poling process. This poling history dependent V_O_ effect on the CST process in a semiconductor photoelectrodes can provide new insights into the application of V_O_ in tuning the optoelectrical feature of semiconductors.

## Results

**Figure 1:**
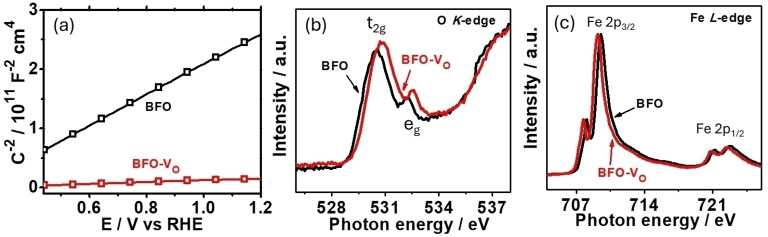
The comparison between BFO (black) and BFO‐V_O_ (red) photoelectrodes in terms of (a) the Mott‐Schottky curves, (b) X‐ray absorption of O *K*‐edge spectra, and (c) X‐ray absorption of Fe *L*‐edge spectra.

The BiFeO_3_ photoelectrode was prepared with previously reported spin coating method, achieving a condensed film with a thickness around 100 nm (Figure S1).^[10]^ The light absorption (Figure S2) and X‐ray diffraction patterns (Figure S3) have demonstrated the formation of well crystallized BiFeO_3_. To induce V_O_, the BiFeO_3_ photoelectrode (BFO) undergoes another annealing treatment at 600 °C in N_2_ atmosphere for 4 hours with the samples noted as BFO‐V_O_ (See Supporting Information for detail). This heat treatment does not show significant change of the light absorption and crystal phase. The UV‐Visible light absorption spectra of the photoanodes display similar band gap of around 2.2 eV before and after N_2_ treatment (Figure S2). Moreover, this treatment has been reported to create oxygen vacancies in other semiconductors and tuning heating duration can tailor the V_O_ concentration.^[29]^ To further check whether this treatment has successfully produced extra V_O_, we first tested the Mott‐Schottky (M‐S) curves. With the generation of V_O_, extra‐charge will be compensated to achieve higher carrier concentration with lower M‐S slope. As shown in Figure 1a, the M‐S slope of BFO‐V_O_ (1.48×10^10^ F^−2^ cm^4^ V^−1^) has been reduced by one order of magnitude compared to BFO (2.58×10^11^ F^−2^ cm^4^ V^−1^), suggesting a much higher V_O_ concentration in BFO‐V_O_. From the intersection of M‐S curves of BFO and BFO‐V_O_ on E‐axial (Figure S4), the flat band potential of them is 0.20 V and 0.22 V vs RHE, respectively.

Furthermore, the X‐ray photoelectron spectroscopy (XPS) has been adopted to show the O 1s chemical states.[^8^, ^9^, ^10^, ^11^, ^29^] With the N_2_ treatment varied from 0 to 4.0 h, the XPS peak area attributes to V_O_ gradually increased, aligned with the change of the carrier concentration in Figure S5. These results further confirm the presence of V_O_ during N_2_ treatment.

To future check the electronic structure change by V_O_, we have investigated the chemical states change of oxygen and iron via near edge X‐ray absorption fine spectroscopy (NEXAFS).[^9^, ^10^, ^11^] According to previous reports,[^30^, ^31^] the formed V_O_ will possess positive charges with the balanced electrons been accepted by the neighbor cations (Fe in BiFeO_3_), wherein the NEXAFS can detect the electron cloud change around the atoms.

From the O *K*‐edge spectra (Figure 1b), it observes a shift towards the high energy for the electrons in t_2g_ and e_g_ orbitals, indicating that O atoms are electron deficient.[^32^, ^33^] As an obvious comparison, the Fe *L*‐edge spectra show a negative shift with the V_O_ existence (Figure 1c), suggesting Fe is in an electron rich status.[^34^, ^35^, ^36^] As a conclusion, the change in the electron cloud around O and Fe atoms clearly evidences the existence of V_O_ in BFO‐V_O_.

**Figure 2:**
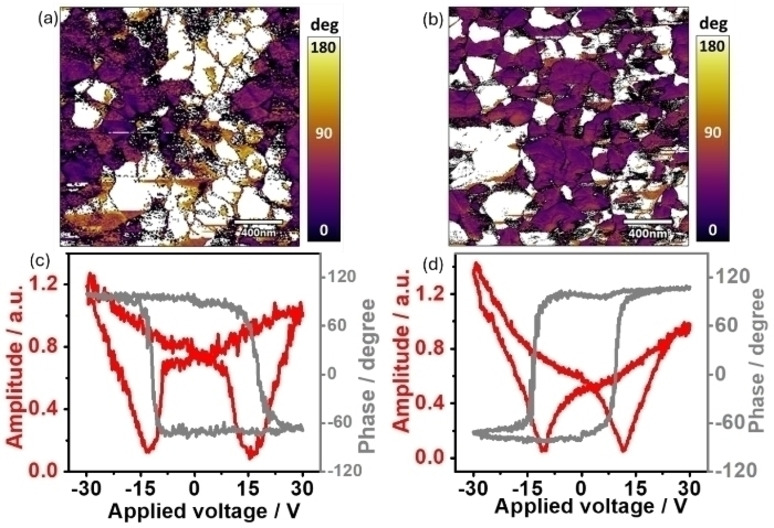
The phase images of (a) BFO and (b) BFO‐V_O_ photoelectrodes. The hysteresis loops of the PFM amplitude and phase for (c) BFO and (d) BFO‐V_O_ photoelectrodes.

With the above characterizations about the structure and electronic status changes, it solidly confirms the generation of V_O_ in the BFO‐V_O_ sample. The presence of V_O_ will interfere with the intrinsic ferroelectric property of BiFeO_3_.[^37^, ^38^, ^39^] The phase images of piezoelectric force microscope (PFM) of BFO and BFO‐V_O_ show that both samples have a 180° phase switch due to the random orientation of the ferroelectric domains (Figure 2 a and b).^[40]^ But the PFM amplitude loop shows significant difference between pristine BFO and BFO‐V_O_. As shown in Figure 2c, a symmetric butterfly loop in the amplitude response is observed in BFO. After introducing V_O_, the PFM amplitude loop is significantly distorted, indicating the presence of defect dipole originated from V_O_ (Figure 2d). Actually, due to the charge compensation, V_O_ will be positively charged and the surrounding cations been negatively charged as revealed in NEXAFS. As a result, defect dipole moments will be established between these positive and negative charges. These defect dipoles can interfere with the intrinsic ferroelectric dipoles in BiFeO_3_, resulting in the distorted butterfly loop in Figure 2d.[^41^, ^42^]

**Figure 3:**
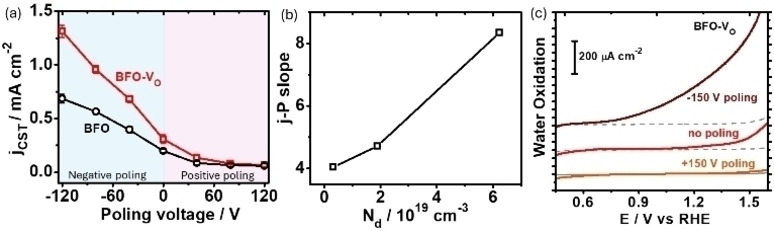
(a) The change of CST capability with the change of external poling voltage. (b) The change of j‐P slope for the negative poling with the change of carrier concentration. (c) The linear sweep voltammetry curves for water oxidation process on BFO‐V_O_ photoelectrodes under −120 V poling, no poling and +120 V poling treatment.

With the BiFeO_3_ photoelectrodes, we investigated the photoresponse under external poling treatment (Figure S6). The CST photocurrent density (j_CST_) is measured to reflect the CST driving force change in the samples.^[43]^ Using the j_CST_ at 1.23 V versus reversible hydrogen electrode (V_RHE_) as a benchmark, the j_CST_‐poling voltage (j‐P) curves are acquired as shown in Figure 3a. Compared to BFO, the j_CST_ of BFO‐V_O_ has improved from 0.20 mA cm^−2^ to 0.31 mA cm^−2^ due to the V_O_ induced CST driving force (that is the V_O_ effect). When the poling voltage continually changes from −120 V to 120 V, it is found that the BFO‐V_O_ show a gradually decreased j_CST_. Especially, when BFO‐V_O_ is poled at −120 V, the j_CST_ will increase to 1.31 mA cm^−2^, five times larger than the original j_CST_ of BFO. In contrast, when poled at +120 V, the j_CST_ of BFO‐V_O_ will reduce to 0.06 mA cm^−2^, which is only one third of the original j_CST_ of BFO. Actually, due to the ferroelectric feature, the j_CST_ of BFO will also be tuned by external poling as shown in Figure 3a. When comparing the BFO and BFO‐V_O_ at same poling voltage, we can conclude that the V_O_ effect is gradually decreased with the poling voltage varied from −120 V to 120 V. With the presence of V_O_, we can observe a more profound change upon external poling treatment. Taking the slope of j‐P as example, under negative poling, the BFO‐V_O_ shows a slope of 8.5 μA cm^−2^ V^−1^, much steeper than that of BFO photoelectrode (4.04 μA cm^−2^ V^−1^). Using the carrier density (N_d_) to reflect the V_O_ concentration change, the relationship between the slope and charge carrier density in Figure 3b further shows the significant role of V_O_ in poling treatment. Under positive poling, both samples experience a gradual photocurrent decay, but the V_O_ makes the j_CST_ decay faster.

The dependence of V_O_ effect on poling treatment can be explained with the defect dipole as discovered in Figure 2. Due to the interference of defect dipole with the intrinsic dipole in BiFeO_3_, it will strengthen the polarization of the semiconductors, leading to enhanced depolarization electric field produced when the dipoles are aligned under external poling.[^19^, ^20^, ^21^, ^22^, ^44^] With negative poling, the depolarization electric field has the same direction as the built‐in electric field for the CST process. Therefore, the adding of defect dipole into the depolarization process will strengthen the CST process, which is reflected as the larger slope of BFO‐V_O_ in j‐P curves. With the generation of more V_O_ in BiFeO_3_, the defect dipole moment will be stronger, resulting in more significant V_O_ effect and steeper j‐P slope at high V_O_ concentration in Figure 3b. Under positive poling, since the depolarization electric field is against the built‐in electric field, the additional defect dipole will further weaken the CST driving force produced by the built‐in electric field. As a result, the presence of V_O_ will reduce the photocurrent more significantly and eventually eliminate V_O_ effect under positive poling.

Moreover, we have investigated the PEC water oxidation to show the influence of poling treatment on V_O_ modified samples. As shown in Figure 3c, we can observe over 100 times improvement of water oxidation photocurrent density (at 1.23 V_RHE_) from 21 μA cm^−2^ to 261 μA cm^−2^ after −120 V poling, which represents as the top photoresponse based on BiFeO_3_ photoelectrodes (Table S1). While poling at +120 V has reduced the photocurrent density to nearly null response, together with significantly improved onset potentials.

**Figure 4:**
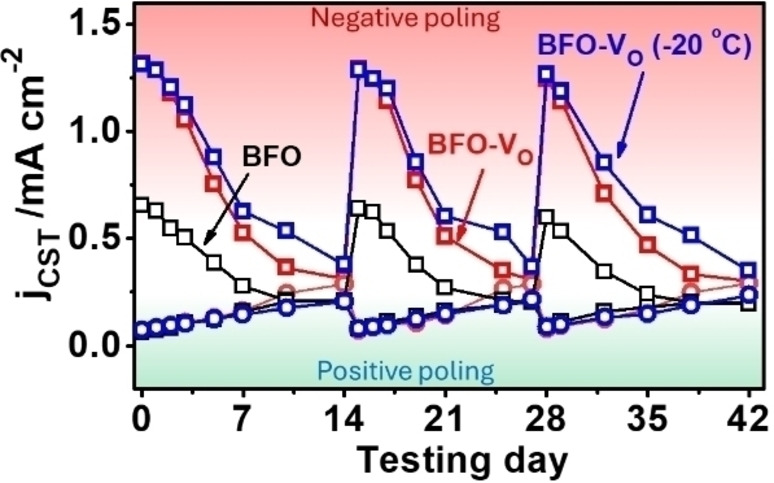
The CST photocurrent density recovery process after negative poling (−120 V, square) and positive poling (+ 120 V, circle). The BFO (black), BFO‐V_O_ (red), and −20 °C freezed BFO‐V_O_ (blue) samples are monitored for comparison.

Actually, all above PEC tests are carried out right after the poling treatment. But the polarization in the BiFeO_3_ is at a metastable status, and relaxation of the aligned dipoles will slowly occur when the poling electric field is perished.^[45]^ To investigate this process, we monitored the j_CST_ of the BiFeO_3_ photoelectrodes over a long period. For both BFO and BFO‐V_O_, they experienced a steady j_CST_ photocurrent change. The negatively poled samples will gradually lose the photocurrent improvement, while positively poled ones slowly recover in a daily based monitoring process, until both samples achieve their original performance after 7 days (Figure 4). More interesting, the effect of polarization can be regained with another same poling stimulation and no significant photocurrent loss is observed with the three rounds poling‐relaxation processes. Furthermore, we can see the BFO‐V_O_ has experienced a very similar recovery rate as the BFO (Figure S7), indicating that the relaxation of polarization process is similar in these two samples. That is reasonable considering that the polarization relaxation is a temperature dependent process, wherein BFO and BFO‐V_O_ are both stored at room temperature. To further check the above assumption, the storage temperature of BFO‐V_O_ has been reduced to −20 °C in a freezer. From Figure 4, we can observe that the photocurrent recovery process has been slowed down, confirming the temperature dependent relaxation process.

The above findings reveal that V_O_ can strengthen the polarization in BiFeO_3_ photoelectrodes, leading to tunable band bending and CST process during PEC research. At negative poling, the V_O_ induced defect dipoles can further strengthen the depolarized electric field established by the ferroelectric dipoles, thereby providing stronger band bending as schemed in Figure S8. Consequently, the V_O_ will promote the CST much more significantly than the non‐polarized BFO samples. While at positive poling, the V_O_ will deteriorate the CST process, leading to even weaker band bending and worse CST performance than the pristine BFO. In addition, the polarization will experience a relaxation process, leading to a dynamic role of the V_O_ induced defect dipole.

## Conclusions

In conclusion, we have investigated the influence of V_O_ on the polarization of typical BiFeO_3_ photoelectrodes. The V_O_ induced defect dipole was found to strengthen CST in ferroelectric BiFeO_3_ under negative poling, while positive poling led to reduced PEC performance. In addition, polarization experienced a slow relaxation process, leading to the decay of the polarization influence. Therefore, the role of V_O_ in a polarized system is drastically determined by the poling history of the materials, including the poling direction, intensity and relaxation time and temperature. These findings enhance our understanding on the nature of V_O_ and shed light on the proper application of V_O_ in tuning optoelectrical behavior of a semiconductor photoelectrode.

## Supporting Information

The experiment details and additional data and references are available within the Supporting Information.

## Conflict of Interests

The authors declare no conflict of interest.

1

## Supporting information

As a service to our authors and readers, this journal provides supporting information supplied by the authors. Such materials are peer reviewed and may be re‐organized for online delivery, but are not copy‐edited or typeset. Technical support issues arising from supporting information (other than missing files) should be addressed to the authors.

Supporting Information

## Data Availability

The data that support the findings of this study are available from the corresponding author upon reasonable request.
