# Supplementary material for: Oxygen Vacancy: How Will Poling History Affect Its Role in Photoelectrocatalysis
Source: ChemSusChem. 2024 Aug 8;17(23):e202400946. doi: 10.1002/cssc.202400946 (PMC11632579; doi:10.1002/cssc.202400946)
Supplement: Supplementary file 1 — Supporting Information [file CSSC-17-e202400946-s001.pdf]

ChemSusChem

Supporting Information

Oxygen Vacancy: How Will Poling History Affect Its Role in Photoelectrocatalysis

Xianlong Li, Zhiliang Wang,* Yifan Bao, Haijiao Lu, Jiakang You, and Lianzhou Wang*

Supporting information

Oxygen Vacancy: How Will Poling History Affect Its Role in Photoelectrocatalysis

Xianlong Li, Zhiliang Wang,* Yifan Bao, Haijiao Lu, Jiakang You, Lianzhou Wang*
Nanomaterials Centre, School of Chemical Engineering, and Australian Institute for Bioengineering and Nanotechnology, The University of Queensland, St Lucia, QLD 4072, Australia
*Email: zhiliang.wang@uq.edu.au; l.wang@uq.edu.au

1. Experimental Section

$BiFeO_3$ photoelectrode fabrication

$BiFeO_3$ (BFO) photoelectrode is synthesized using a spin coating method.[1] Typically, 0.5 M $Bi(NO_3)_3·5H_2O$ (Sigma-Aldrich, 98%) and $Fe(NO_3)_3·9H_2O$ (Sigma-Aldrich, 98%) were dissolved in 2-methoxyethanol ($CH_3OCH_2CH_2OH$, Sigma-Aldrich, 99.8%) and acetic acid (CH_3CO_2H, Sigma-Aldrich, 99.7%) mixture solution. The deposition of BFO film was carried out by spin coating of the gel precursor on the FTO substrate with the high-speed rotation of 3000 rpm for the 30s to form thin liquid films. This deposition process was repeated for 5 times and then pre-annealed on a hot plate at 350 ^oC for 15 min, followed by further calcinating at 700 °C for two hours.

The introduction of oxygen vacancies on BFO photoelectrode was conducted by calcinating the as-prepared BFO photoelectrode in a pure nitrogen flow at 600 °C (2 °C/min) for 0, 2, 4 hours in a tube furnace. The 4-hour-N_2-treated sample was denoted as BFO-V_O.

Poling treatment of the BFO film

Poling treatment was carried out with home-made device in Figure S4 with a DC power source (ITECH DC POWER SUPPLY, IT6834). The Ti plate was covered by the kapton tape for electrical insulation and supported by the conductive needle connected to the DC power source. A DC voltage of 0~120 V is loaded between film

photoelectrode and the Ti plate electrode in dark for 600s. The bias loaded on $BiFeO_3$ is referred to Ti, i.e., when $BiFeO_3$ is connected to the positive pole and Ti to the negative pole of the DC power source, the poling voltage is noted as positive.

Structure characterization

The morphology of $BiFeO_3$ photoelectrode was imaged with scanning electron microscope (SEM, JEOL JSM 7001F). The film thickness of the photoelectrode was measured by cross-section SEM imaging. The crystal phase structure is measured by X-ray diffraction spectroscopy (XRD, Bruker D8 Advanced diffractometer) with Cu Kα (λ=1.5418 Å) radiation. The optical absorbance spectrum is obtained by the ultraviolet–visible (UV–Vis) absorption spectra (JASCOV-650 spectrophotometer). The X-ray absorption structure (XAS) information over the Fe L-edge and O K-edge of the $BiFeO_3$ photoelectrode was collected at the Australian Synchrotron. The element chemical state is analysed with X-ray photoelectron spectroscopy (XPS, Kratos Ultra) with a mono Al X-ray source.

Photoelectrochemical and Electrochemical measurements

Photoelectrochemical performance (photocurrent density) and Mott–Schottky measurements were tested on the 3-electrode system with CHI660E potentiostat. The as-synthesized $BiFeO_3$ photoelectrode acted as working electrode combined with Pt wire and saturated calomel electrode (SCE) as the counter and reference electrodes, respectively. Xenon lamp with an AM 1.5G filter (Newport) is used as light source for test. The light intensity is calibrated to be 100 mW cm^{-2} with a standard Si-diode. The water oxidation measurement was conducted in the aqueous electrolyte composed of 1.0 M NaOH (Sigma-Aldrich, 97%) solution (pH 13.6). The charge separation and transfer efficiency were tested in the aqueous solution of 0.1 M H_2O_2 (Merck, 30%) and 1M NaOH mixture with the photocurrent noted as j_{CST}. The linear scan voltammetry method is applied for the test with a voltage range from -0.6~ 0.7 V vs reversible hydrogen electrode (RHE). Mott-Schottky (M-S) curves were measured at a frequency of 1 kHz from -0.6V to 0.7V in 1.0 M NaOH buffer (~pH 13.6). The measured potential vs. SCE was converted to RHE scale using the Nernst equation:

$$E_{RHE} = E_{SCE} + 0.059 \times pH + 0.2411$$

Based on the M-S curves, the charge carrier density (Nd) was calculated using the following equation[8]:

$$Nd = \frac{2}{e\varepsilon_0\varepsilon} \times \left(\frac{dC^{-2}}{dV_{RHE}}\right)^{-1}$$

Where the electronic charge (e) is 1.6 × 10^{-19} C, vacuum permittivity (ε_0) is 8.85 × 10^{-14} Fm^{-1}, and relative permittivity (ε) is 175 for BFO.[2] C (Fcm^{-2}) is the space charge capacitance in the semiconductor (obtained from the slope of MS curves), and V_{RHE} is the applied potential for MS curves.

Characterization of ferroelectric property

Microscopic ferroelectric property of the $BiFeO_3$ photoelectrode were identified by piezoresponse force microscope (PFM) pattern.[3] PFM measurements were also performed on the same atomic force microscope (Cypher, Asylum Research) under ambient conditions at room temperature. The conductive Pt-coated Si probe (Olympus ASYELEC.01-R2, spring constant ~2.8 N/m and tip radius ~28 nm) was used for the study of domain imaging and polarization switching.

2. Supporting figures

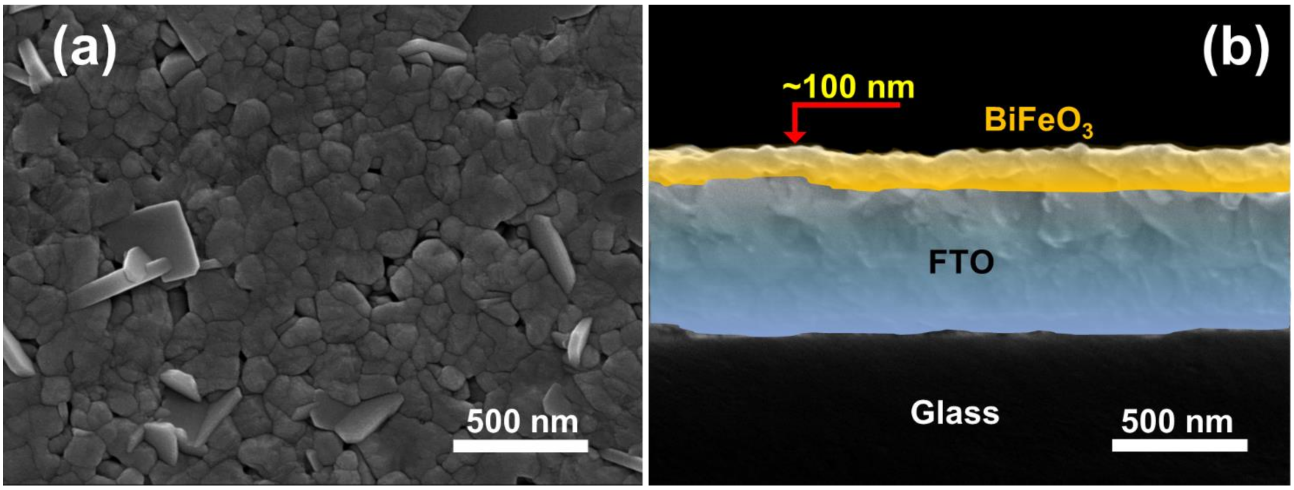

Figure S1. (a) The top-view and (b) cross section images of as prepared BFO photoelectrode.

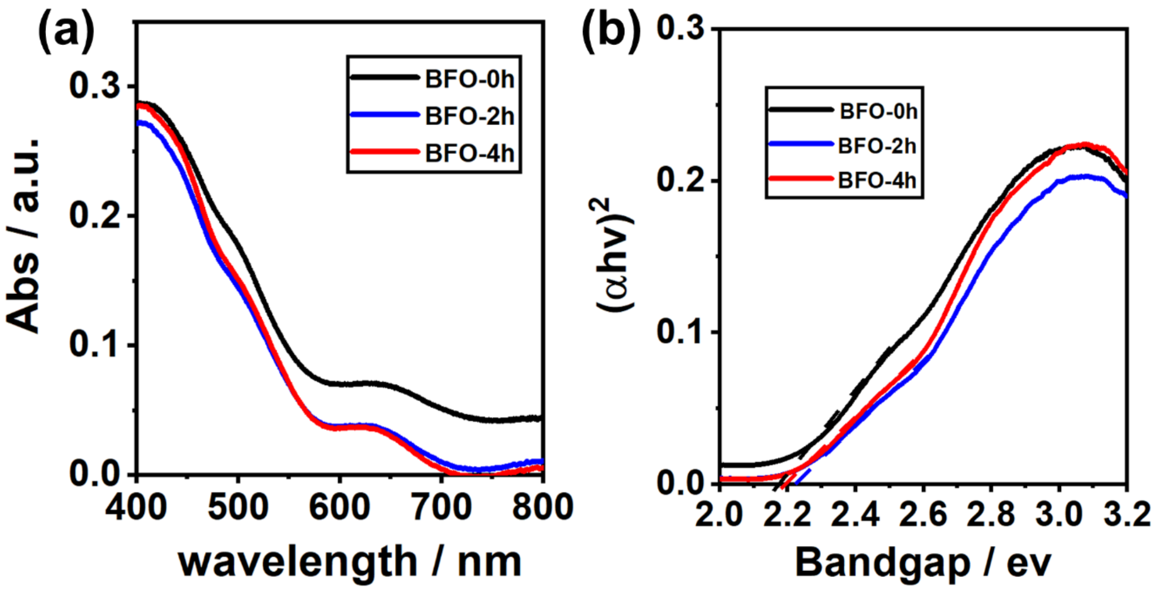

Figure S2. (a). UV-vis light absorption curves and (b). Tauc plot of the BFO treated in N_2 for different periods of time (0–4.0h).

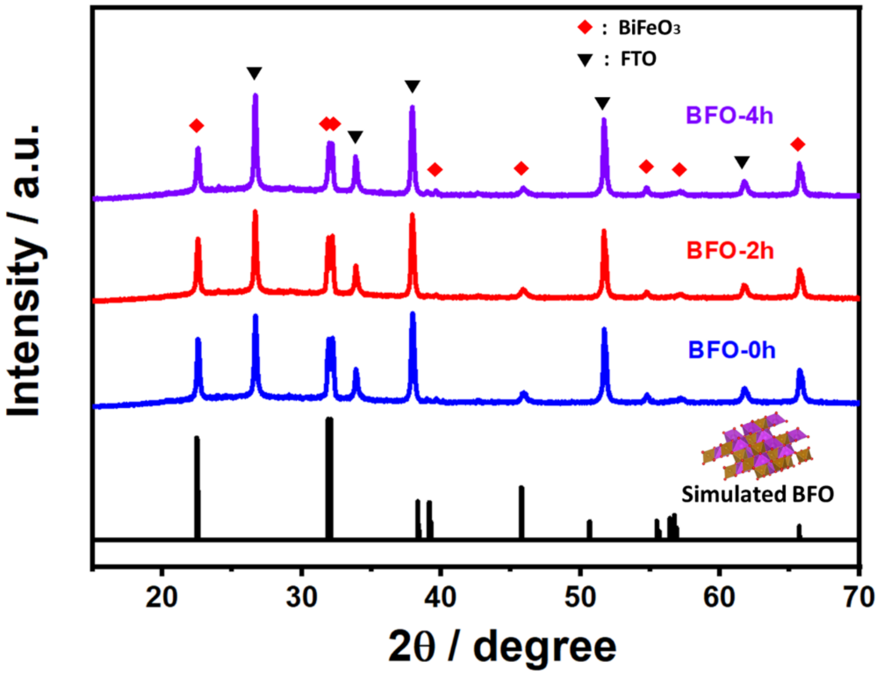

Figure S3. The XRD comparison of as-prepared BFO photoelectrode with the BFO after N_2 treatment, respectively.

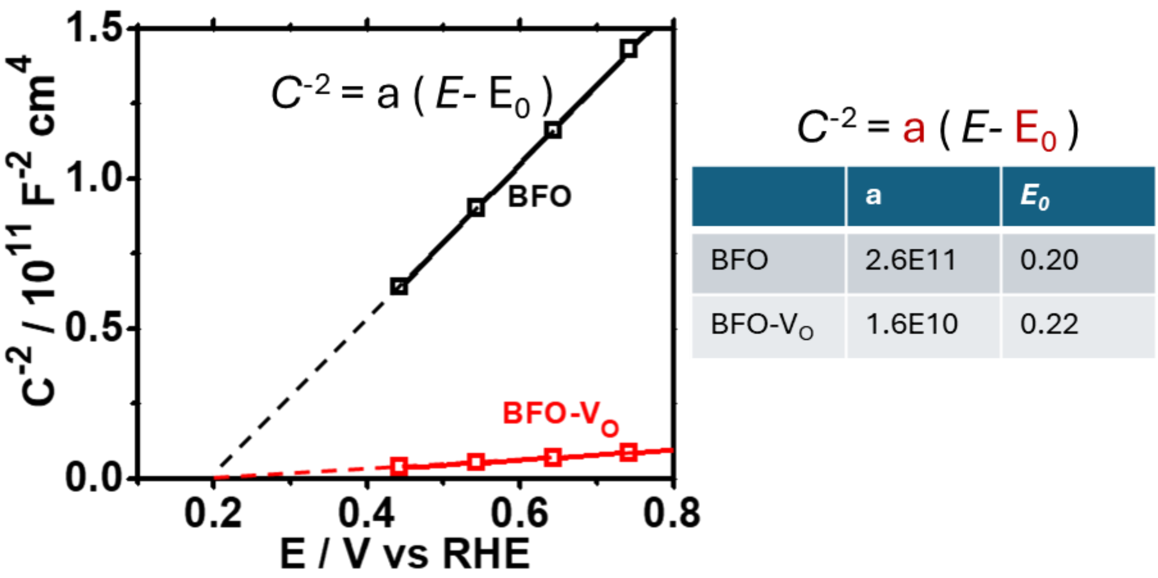

$C^{-2} = a (E - E_0)$

	a	E_0
BFO	2.6E11	0.20
BFO-V_O	1.6E10	0.22

Figure S4. The Mott-Schottky curves of BFO and BFO-VO, together with the fitting of the curves to show the slope (a) and intersection (E_0).

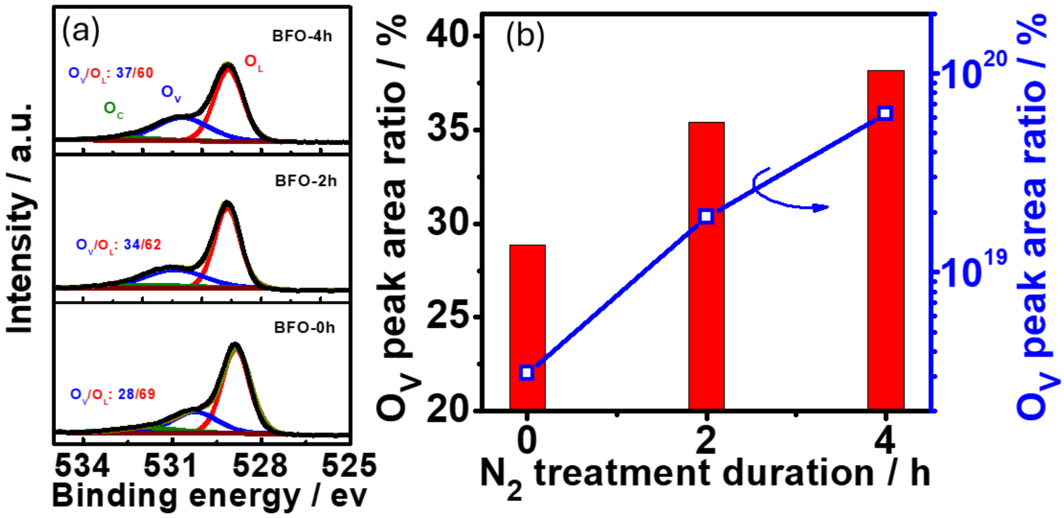

Figure S5. The high-resolution XPS (c) O1s spectra for BFO treated in N_2 for different duration (0–4.0 h). (b) The O_v peak area percentage (cyan/orange bars) and the carrier concentration for BFO treated in N_2 for different duration (0–4.0 h).

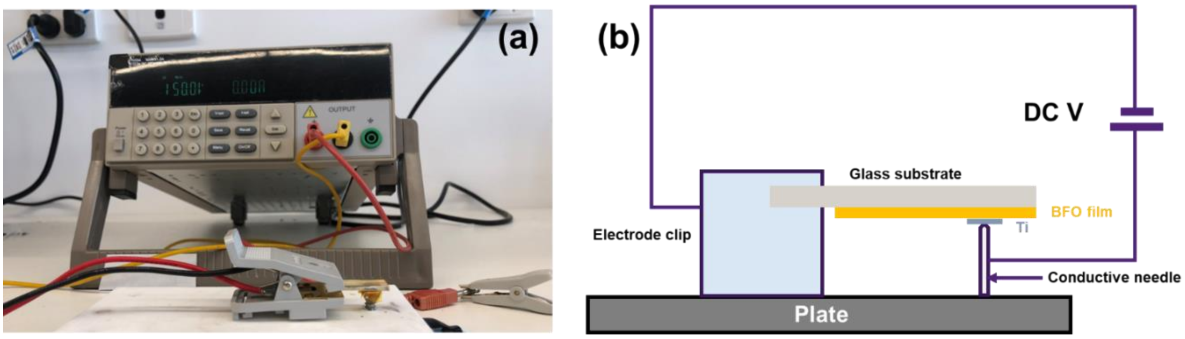

Figure S6. (a) The setup for poling treatment and (b) schematic image of home-made device for negative poling treatment

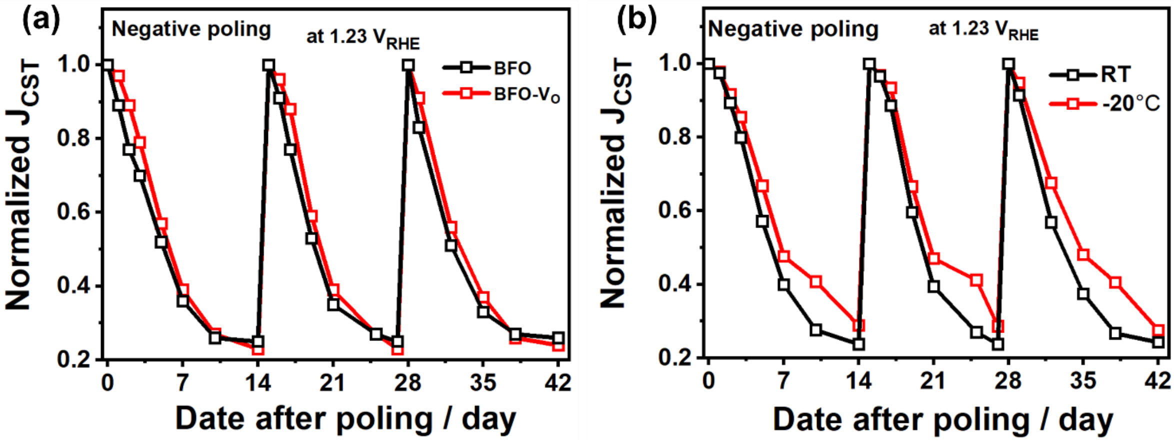

Figure S7. Normalized CST capability fortnight after negative poling for (a) BFO (black) and BFO-V_O (red) samples stored in room temperature (RT) and (b) BFO-V_O stored in room temperature (black), and -20 ^oC (red).

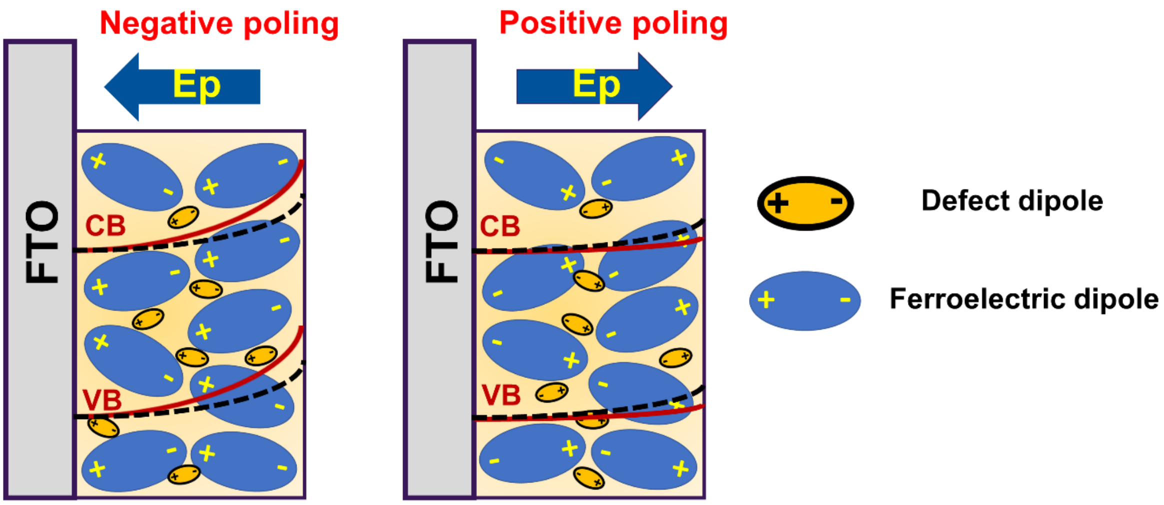

Figure S8. The schematic illustration about how VO induced defect dipoles interact with intrinsic ferroelectric dipoles in $BiFeO_3$ photoelectrodes.

Table S1. The comparison of photocurrent based on BFO photoelectrodes.

	Sample name	Photocurrent	reference
1	BFOv-(-120V)	420 μA cm^{-2} @ 1.4 V vs RHE	Our work
2	BFO-(-40V)	88.7 μA cm^{-2} @ 1.4 V vs RHE	Our previous work, ACS Nano 2023, 17, 22944
3	BFO-(-8V)	8 μA cm^{-2} @ 0 V vs Ag/AgCl	Angew. Chem. Int. Ed. 2014, 53, 11027 - 11031
4	CoOx/TiO_2 / BFO	200 μA cm^{-2} @ 1.4 V vs RHE	Solar Energy, 2020, 202, 198
5	LPCVD-grown BFO	160 μA cm^{-2} @ 1.4 V vs RHE	Nanoscale, 2015, 7, 16343
6	BFO	24 μA cm^{-2} @1.4 V vs RHE	Int. J. Electrochem. Sci., 2021, 16, 210721
7	poled super-domain BFO-OH	600 μA cm^{-2} @ 1.4 V vs RHE (single crystal)	Adv. Funct. Mater., 2022, 32, 2111180.

Reference List

[1] X. Li, Z. Wang, W. Ji, T. Lu, J. You, K. Wang, G. Liu, Y. Liu, L. Wang, *ACS Nano,* **2023**, *17*, 22944.

[2] A. Blázquez Martínez, N. Godard, N. Aruchamy, C. Milesi-Brault, O. Condurache, A. Bencan, S. Glinsek, T. Granzow, *J. Eur. Ceram. Soc.,* **2021**, *41*, 6449.

[3] a) Y. Liu, S. Ye, H. Xie, J. Zhu, Q. Shi, N. Ta, R. Chen, Y. Gao, H. An, W. Nie, H. Jing, F. Fan, C. Li, *Adv. Mater.,* **2020**, *32*, 1906513; b) S. Gupta, M. Pal, M. Tomar, R. Guo, A. Bhalla, V. Gupta, *J. Alloys Compds.,* **2021**, *882*.
